# Successfully REBOA performance: does medical specialty matter? International data from the ABOTrauma Registry

**DOI:** 10.1186/s13017-020-00342-z

**Published:** 2020-11-23

**Authors:** Peter Hilbert-Carius, David McGreevy, Fikri M. Abu-Zidan, Tal M. Hörer, The ABOTrauma Registry research group, M. Sadeghi, A. Pirouzram, A. Toivola, P. Skoog, K. Idoguchi, Y. Kon, T. Ishida, Y. Matsumura, J. Matsumoto, M. Maszkowski, A. Bersztel, E. C. Caragounis, T. Bachmann, M. Falkenberg, L. Handolin, S. W. Chang, A. Hecht, B. Kessel, D. Hebron, G. Shaked, M. Bala, F. Coccolini, L. Ansaloni, R. Hoencamp, Yunus Emre Özlüer, T. Larzon, K. F. Nilsson

**Affiliations:** 1 Department of Anesthesiology, Intensive Care and Emergency Medicine, Bergmannstrost Hospital Halle, Mersbuegerstraße 165, 06112 Halle (Saale), Germany; 2 Department of Cardiothoracic and Vascular Surgery, Faculty of Medicine and Health, Örebro University, Örebro, Sweden; 3 Department of Surgery, College of Medicine and Health Science, UAE University, Al-Ain, United Arab Emirates; 4 Department of General Surgery, Faculty of Medicine and Health, Örebro University, Örebro, Sweden

**Keywords:** Resuscitative endovascular balloon occlusion of the aorta, Trauma, Bleeding, Performance, Registry

## Abstract

**Background:**

Resuscitative endovascular balloon occlusion of the aorta (REBOA) is a minimally invasive procedure being increasingly utilized to prevent patients with non-compressible torso hemorrhage from exsanguination. The increased use of REBOA is giving rise to discussion about “Who is and who should be performing it?”

**Methods:**

Data from the international ABO (aortic balloon occlusion) Trauma Registry from between November 2014 and April 2020 were analyzed concerning the question: By who, how, and where is REBOA being performed? The registry collects retrospective and prospective data concerning use of REBOA in trauma patients.

**Results:**

During the study period, 259 patients had been recorded in the registry, 72.5% (*n* = 188) were males with a median (range) age of 46 (10-96) years. REBOA was performed in the ER in 50.5%, in the OR in 41.5%, and in the angiography suite in 8% of patients. In 54% of the patients REBOA was performed by surgeons (trauma surgeons 28%, vascular surgeons 22%, general surgeons 4%) and in 46% of the patients by non-surgeons (emergency physicians 31%, radiologists 9.5%, anesthetists 5.5%). Common femoral artery (CFA) access was achieved by use of external anatomic landmarks and palpation alone in 119 patients (51%), by cutdown in 57 patients (24%), using ultrasound in 49 patients (21%), and by fluoroscopy in 9 patients (4%). Significant differences between surgeons and non-surgeons were found regarding patient’s age, injury severity, access methods, place where REBOA was performed, location patients were taken to from the emergency room, and mortality.

**Conclusion:**

A substantial number of both surgical and non-surgical medical disciplines are successfully performing REBOA to an almost equal extent. Surgical cutdown is used less frequently as access to the CFA compared with reports in older literature and puncture by use of external anatomic landmarks and palpation alone is used with a high rate of success. Instead of discussing “Who should be performing REBOA?” future research should focus on “Which patient benefits most from REBOA?”

**Supplementary Information:**

The online version contains supplementary material available at 10.1186/s13017-020-00342-z.

## Background

Resuscitative endovascular balloon occlusion of the aorta (REBOA) is a minimally invasive procedure being increasingly utilized to prevent patients with non-compressible torso hemorrhage (NCTH) from exsanguination. It is used as a bridge to surgical bleeding control to gain time during the management of hemorrhagic shock as part of the endovascular resuscitation and trauma management (EVTM) concept [1–7]. Access to the common femoral artery (CFA) is essential for performing REBOA and is regularly gained by different medical specialists including vascular surgeons, interventional radiologists, anesthetists, and emergency physicians [8]. Many medical specialists, therefore, have the necessary skill to perform REBOA in NCTH to temporarily diminish ongoing hemorrhage with the aim of saving the life of the patient. In very urgent cases, REBOA is required within the first few minutes of patient arrival. Traditionally, not all “potential REBOA” medical disciplines are involved in the emergency room (ER) management of trauma patients, and often only trauma and/or general surgeons participate on a regular basis, depending on the local trauma team structure and setting. On the other hand, REBOA is not only performed in the ER but also in the operating room (OR), angiography suite (AS), and occasionally in the pre-hospital setting; it is also used for some causes of non-traumatic hemodynamic instability [9–11]. Following the publication of the joint statement from the American College of Surgeons’ Committee on Trauma (ACS-COT) and the American College of Emergency Physicians (ACEP) addressing the clinical use of REBOA, the question of “Who should be the one in charge of REBOA” began to be discussed [12–16].

With the present study, we aimed to describe by who, how (regarding the access), and where REBOA is successfully being performed, using data from the unique international ABO (aortic balloon occlusion) Trauma Registry. We believe that this is highly relevant since the indications for the use of REBOA are also starting to include non-traumatic causes of hemodynamic instability or even cardiopulmonary resuscitation (CPR) [2, 17].

## Method

Data from the ABOTrauma Registry between November 2014 and April 2020 were analyzed. The ABOTrauma Registry collects retrospective and prospective data concerning the use of REBOA in trauma patients in hemorrhagic shock [18]. Patients from 22 centers in 13 countries were included. Currently, the ABOTrauma Registry is the only registry with international REBOA data from four continents (Europe, Asia, Africa, and South America). Center recruitment is voluntary, with known REBOA-practicing institutions being invited to participate. Centers can also register independently via the registry website after approval from the principal investigators. There are no center-specific criteria such as minimum case volume or hospital size. All participating centers were prepared for the REBOA procedure in advance by different courses and underwent some form of additional training and familiarization with REBOA access and devices prior to employing them. The ABOTrauma Registry only includes patients with successfully placed REBOAs and does not capture data of failed REBOA attempts. The registry is funded and hosted by the Department of Cardiothoracic and Vascular Surgery, Örebro University Hospital, Sweden. Ethical approval for the registry was obtained from the regional committee (study number: 2014/210; Regionala Etikprövningsmyndigheten, Uppsala, Sweden). Patient data are anonymized at the point of registration through the use of a unique registry-generated ID number. No patient identifiable data (name, hospital number, date of birth) are held in the registry, which is in line with the current European general data protection regulation. The need for ethical approval of the current study was waived by the ethical committee of the Medical Association Saxony-Anhalt Germany (ID-Nr. 80/19).

### Statistical analysis

The statistical analysis aimed to answer the question “By who, how, and where is REBOA performed?” Characteristics of the treated patients as well as REBOA-specific issues, such as access method and place where REBOA was performed, were compared for surgeons and non-surgeons.

Data were presented as median (range) for ordinal and continuous data, and number (%) for categorical data. If data were missing, valid percentages were calculated from the available data. We used non-parametric statistical methods because of the small sample size of the groups. These methods do not need a normal distribution because they analyze the ranks and not the crude numbers. Categorical data of two independent groups were analyzed using Pearson’s chi-square or Fisher’s exact test as appropriate. Overall significance was tested for large tables, and if the overall analysis was significant, pairwise comparisons were performed to explain the location of the significance. Mann-Whitney *U* test was used to compare continuous or ordinal data for two independent groups. Statistical analyses were performed using the Statistical Package for the Social Sciences (IBM-SPSS version 26, Chicago, Il). A *p* value of less than 0.05 was considered significant.

## Results

### Demographics

During the study period, 259 patients had been recorded in the registry; 72.5% (*n* = 188) were males with a median (range) age of 46 (10-96) years. Demographic data of the study population are summarized in Table 1. The majority (74.5%) of patients sustained blunt trauma (*n* = 193), 22.7% penetrating trauma (*n* = 59), five patients (1.9%) a combination of blunt and penetrating trauma, and in two cases (0.5%) the trauma sustained was unknown. Head injury was present in 80 patients (31%).

**Table 1:** General data regarding vital signs and injury severity

	Median (range)	25-75% percentile	Mean (± SD)	95% CI
Age (years) *n* = 248	46 (10-96)	27-61.75	46.46 (± 20.78)	43.83-49.01
ISS (points) *n* = 206	38 (11-75)	25-50	40.18 (± 16.32)	37.95-42.40
Sys. BP at scene (mmHg) *n* = 218	50 (0-133)	10-75	48.95 (± 38.11)	43.86-54.04
GCS at scene (points) *n* = 172	11 (3-15)	4-14	9.55 (± 4.66)	8.85-10.25
SpO_2_ on admission (%) *n* = 168	90 (50-100)	78-96	85.9 (± 12.3)	84.11-87.86
HR on admission (bpm) *n* = 226	120 (0-145)	95-130	107 (± 35.11)	102.4-111.6
Sys. BP on admission (mmHg) *n* = 248	60 (0-155)	35-79.75	58.19 (± 34.5)	53.33-62.51
Sys. BP before ABO (mmHg) *n* = 243	52 (0-150)	40-70	49.26 (± 31.13)	45.33-53.20
Sys. BP after ABO (mmHg) *n* = 243	97 (0-203)	80-115	95.7 (± 39.4)	90.73-100.7

*ISS* Injury Severity Score, *sys. BP* systolic blood pressure, *GCS* Glasgow Coma Scale, *HR* heart rate, *SpO*_*2*_ oxygen saturation, *ABO* aortic balloon occlusion

### CFA access data

Data concerning the side of access to the CFA was available for 188 patients. CFA access was achieved on the right side in 126 patients (67%), on the left-hand side in 48 patients (25.5%), and bilaterally in 14 (7.5%) patients. Data concerning the method of gaining access were present for 234 patients. CFA access was performed with the use of external anatomic landmarks alone (percutaneous puncture guided by palpation and external landmarks but without ultrasound or fluoroscopy) in 119 patients (51%), by cutdown in 57 patients (24%), using ultrasound in 49 patients (21%), and by fluoroscopy in nine patients (4%).

CFA access was achieved at the first attempt in 138 patients (53%), by the second or third attempt in 62 patients (24%), and by more than three attempts in 12 patients (5%). For 47 patients (18%), the number of attempts was unknown.

### Location

Information regarding the physical location where REBOA was performed was available for 243 patients. REBOA was carried out in the ER in 123 patients (50.5%), in the OR in 101 patients (41.5%), and in the AS in 19 patients (8%).

### Complications

The overall rate of complications, including minor and major complications was 13.8% (*n* = 36). Major complications occurred in 8.5% of the patients and included extremity ischemia (*n* = 16), perforation of the aorta or iliac artery (*n* = 4), and massive bleeding in the access site (*n* = 2). Renal failure was present in 27 patients (10.5%) but since all patients were in hemorrhagic shock, it is unclear whether the renal failure was caused by REBOA, shock, or both.

### Who performed REBOA?

Information on “Who performed REBOA?” was present for 248 patients. REBOA was performed by surgeons in 133 patients (54%) and by non-surgeons in 115 patients (46%). Table 2 compares patient characteristics, method of access, access attempts, and the location where REBOA was performed for the surgeons vs non-surgeons group. Significant differences were found in patients’ age, injury severity, access methods, place where REBOA was performed, place where patients were taken from the ER, and mortality. No difference was found regarding the access attempts and in the majority of patients, the access was achieved in 1 to 3 attempts (74.5% surgeon vs 73% non-surgeon). Likewise, there was no significant difference in time from admission to REBOA between surgeons and non-surgeons, although non-surgeons were in median a little faster than surgeons (see Table 2). REBOA was performed by emergency (EM) physicians in 76 patients (31%), by trauma surgeons in 70 patients (28%), by vascular surgeons in 54 patients (22%), by radiologists in 24 patients (9.5%), by anesthetists in 14 patients (5.5%), and by general surgeons in 9 patients (4%).

**Table 2:** Comparison of patient characteristics for surgeons and non-surgeons performing REBOA

	Surgeons (*n* = 133)	Non-surgeons (*n* = 115)	***P*** value
**Age** median (range)	34 (10-90)	58 (10-96)	< 0.001
**Male sex**	102 (76.5%)	78 (68%)	0.153
**ISS** median (range)	32 (14-75)	41 (11-75)	0.011
**Head injury**	32 (24%)	42 (36.5%)	0.47
**CPR at scene**	12 (9%)	20 (17.5%)	0.09
**CPR on admission**	14 (10.5%)	15 (13%)	0.54
**24 h mortality**	33.5%	44.5%	0.08
**30 days mortality**	44.5%	62%	0.006
**REBOA complications**	12.8%	19%	0.22
**Time to REBOA** (min) median (range)	150 (3-1437) (*n* = 92)	85 (0-1426) (*n* = 74)	0.999
**CFA access**			< 0.0001
Blind	39 (29.3%)	79 (68.7%)	< 0.001
Ultrasound	21 (15.8%)	24 (20.9%)	0.322
Cutdown	54 (40.6%)	1 (0.9%)	< 0.001
Fluoroscopy	2 (1.5%)	6 (5.2%)	0.149
Unknown	17 (12.8%)	5 (4.3%)	0.024
**CFA access attempts**			0.504
1	72 (54%)	51 (44.5%)	
2-3	27 (20.5%)	33 (28.5%)	
> 3	10 (7.5%)	2 (1.75%)	
Unknown	24 (18%)	29 (25.25%)	
**REBOA place**			< 0.0001
ER	60 (45.1%)	60 (52.1%)	0.27
OR	69 (51.9%)	29 (25.2%)	< 0.001
AS	3 (2.3%)	14 (12.2%)	0.002
Unknown	1 (0.8%)	12 (10.4%)	< 0.0001
**Patients transferred to**			< 0.0001
OR	90 (67.7%)	47 (40.9%)	< 0.0001
CT	16 (12%)	14 (12.2%)	0.999
AS	5 (3.8%)	23 (20%)	< 0.0001
ICU	8 (6%)	1 (0.9%)	0.04
Unknown	14 (10.5 %)	30 (26.1%)	0.0015

*ISS* Injury Severity Score, *CPR* cardiopulmonary resuscitation, *CFA* common femoral artery, *ER* emergency room, *OR* operating room/theater, *AS* angiography suite, *CT* computer tomography, *ICU* intensive care unit

*P* Pearson chi-square, Fisher’s exact test, or Mann-Whitney *U* test as appropriate

The comparisons between the different surgical and non-surgical specialties are shown in the supplemental material of this paper. Table S1 shows specialty performing REBOA, where it was performed and the demographic of patients treated. Table S2 shows specialty performing REBOA and how it was performed.

Non-surgical disciplines such as EM physicians, anesthetists, and radiologists use non-invasive access methods (use of external anatomic landmarks and palpation alone, ultrasound, fluoroscopy) significantly more often compared to surgical disciplines who use cutdown more often to achieve access to the CFA (*p* < 0.001). Surgeons performed REBOA significantly more frequently in the OR compared with non-surgeons (*p* < 0.001). Regarding the potential complications, the difference between surgeons (12.8%) and non-surgeons (19%) was not statistically significant (*p* = 0.22).

## Discussion

**Fig. 1:**
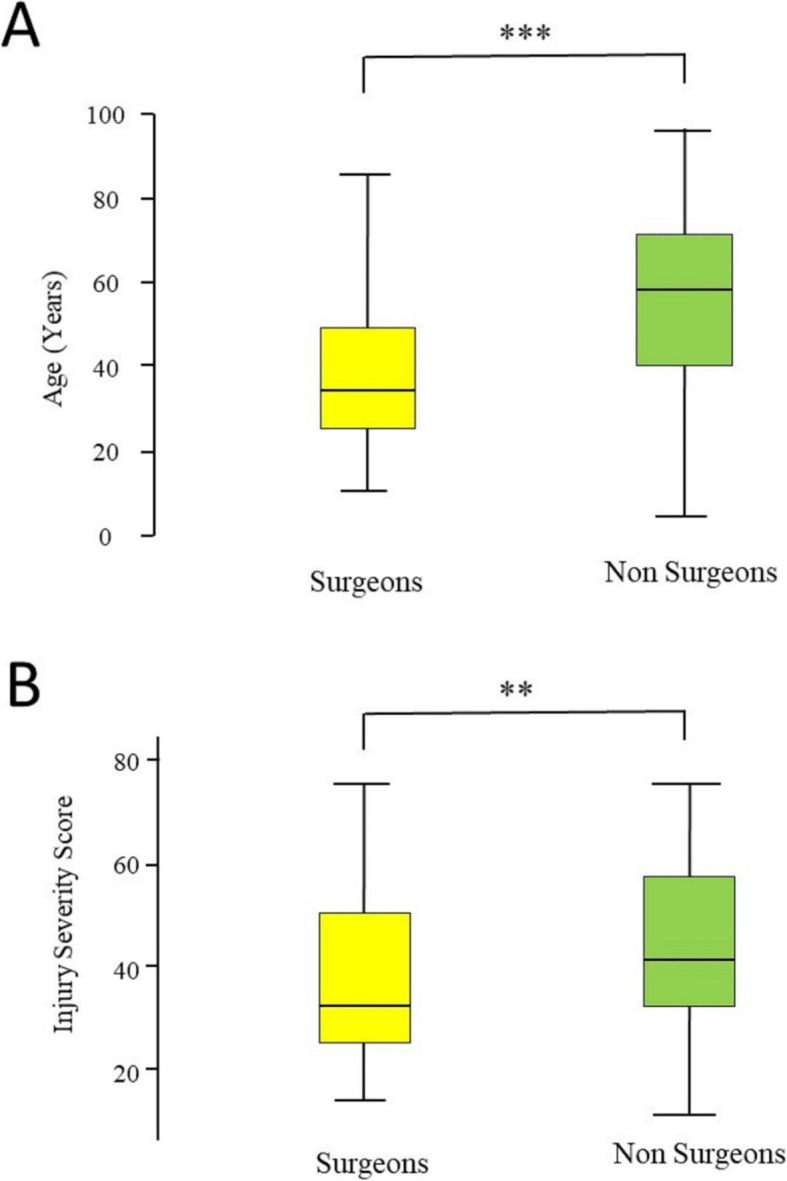
Box-and-whiskers plot of age (**a**) and Injury Severity Score (ISS) of patients who had REBOA performed by surgeons (*n* = 133) and non-surgeons (*n* = 115). The box represents the 25th to the 75th percentile IQR. The horizontal line within each box represents the median. *** = *p* < 0.0001, Mann-Whitney *U* test, ** = *p* < 0.02, Mann-Whitney *U* test

REBOA is being used more frequently as part of the EVTM concept of using minimally invasive methods of resuscitation and bleeding control. With increased use of REBOA, there is increasing debate about “who should be performing REBOA?” [12–16]. This study shows that both surgical and non-surgical physicians are successfully performing REBOA, with it being performed mainly by EM physicians followed by trauma and vascular surgeons. However, there were significant differences in patient characteristics between the two groups. Patients treated by non-surgeons were older and more severely injured. The explanation for these differences is not yet clear and could be multifactorial. One reason could be that in some participating centers, surgeons may not be continuously involved in the management of trauma patients in the ER, for example, the majority of Japanese ERs do not have trauma surgeons [19]. EM physicians may therefore often take the lead in urgent trauma situations with severe injuries. EM physicians usually treat a wide range of patient age groups and diseases. In some countries, trauma surgeons usually treat adult patients, often without associated comorbidities, although this is now changing. In the settings where no surgeons are involved in the ER management and REBOA procedures are performed by surgeons in the OR when needed, patients who arrive to the OR need to survive the ER stage before being treated by surgeons. This might be one reason why patients with REBOA performed by surgeons are less severely injured, younger (see Fig. 1), and have a lower mortality. Another probable reason, as confirmed by our results, is that surgeons who are available in the ER may decide quickly to transfer the patient to the OR for surgery. This reduces the time the patient spends in the ER. There is clear evidence that a short time to definitive bleeding control reduces mortality [20, 21]. The lower mortality in the surgeons group should not be overrated, since this is the unadjusted mortality and, due to the younger age and lower ISS of patients, a higher rate of survival is to be expected. As known from different trauma scores and data from the TraumaRegister DGU® (the trauma registry of the German Trauma Society) injury severity and age over 55 years are significant and strong predictors of mortality [22–25]. The same applies to the lower rate of overall complications in the surgeons group, which could be caused by the younger age, the lower ISS, less comorbidity, or rather by more experiences in invasive measures, early addressing potential complications and their treatment. These data should, therefore, be cautiously interpreted in the discussion concerning “Who should be performing REBOA?”

Independent of the medical specialties performing REBOA, all centers participating in the ABOTrauma Registry were prepared before treating the first patient with REBOA. The preparation phase was almost similar in most centers including:

- Organized workshops prior to initialization of the procedure
- Familiarization with the equipment and developing ultrasound skills specifically to be able to identify the femoral vessels and to perform ultrasound-guided insertion of the needle
- Theoretical presentations on issues regarding REBOA such as when, where, and how to perform the procedure, pitfalls, and complications
- Several centers attended the REBOA/EVTM workshop of the University of Örebro (Sweden)
- Some centers use commercially available REBOA simulators (like the RATT Pulsatile Simulator, REBOA Access Task Trainer) to coach the staff and keep a high-performance level

Regardless of the medical specialty or the trauma center performing REBOA, a key step to successfully save trauma victims is proper training, being prepared with knowledge and continuous education, and being familiar with equipment and setting [26–29]. There is no shortcut to success and “You don’t rise to the occasion, you sink to the level of your training!” (Archilochus—Greek lyric poet) and that is regardless of the medical specialty.

On the other hand, many medical specialties in our study have already more or less daily practice in establishing access to the common femoral artery (e.g., anesthetists, emergency physicians, interventional radiologists, vascular surgeons) and therefore have the tactile practice needed to performing REBOA. But even these medical specialties with experience in gaining common femoral artery access need to be trained in the equipment used (different introducer sheath, REBOA catheter with or without guidewire, pressure monitoring). The participating centers have to take the responsibility to train their staff and keep a high-performance level.

EM physicians, anesthetists, surgeons (trauma, vascular, general), and interventional radiologists can be involved in the immediate and direct care of trauma patients, depending on the setting and trauma team procedures. EM physicians and/or anesthetists control airways, respiration, and hemodynamics to gain time and allow a definitive procedure to be performed by surgeons or interventional radiologists to stop the bleeding [19]. EM physicians, anesthetists, vascular surgeons, and interventional radiologists routinely place sheaths, a technique that is necessary in REBOA. It is, therefore, not surprising that REBOA is performed by all these various medical disciplines. In contrast, other than in Japan, the majority of studies have reported that REBOA was performed at level 1 trauma centers by trauma surgeons who were trained in REBOA or who were experienced in vascular surgery [30–32]. Surgical exposure of the CFA is an essential skill needed for the successful use of REBOA if percutaneous access fails [4]. Early American clinical experience demonstrated that almost half of the patients required surgical cutdown for vascular access. With increased experience over time, subsequent studies have shown more successful percutaneous access [33, 34]. Our study, similar to others, has shown that surgical cutdown is used less frequently and that use of external anatomic landmarks and palpation alone to puncture the CFA has a high rate of success [3]. This could be related to the availability of smaller and guidewire-free REBOA catheters such as the ER-REBOA™ catheter (Boerne, TX, USA), which is compatible with many 7 Fr sheaths. Emergency physicians and anesthetists are familiar with percutaneous ultrasound-guided CFA cannulation, which can avoid the need for surgical cutdown [4]. The reduced profile of new devices will likely increase the use of REBOA by non-surgeons. This is because REBOA has a wide range of possible indications besides trauma, such as cardiac arrest, post-partum hemorrhage, non-trauma-related intra-abdominal hemorrhage, and trauma in the pre-hospital setting [3, 4, 9, 10, 35–37]. In addition, closure after a low profile REBOA device is possible either with percutaneous technique or just pressure bandage, making open surgical techniques for introducer removal optional.

In this study, REBOA was performed in the ER in about 50% of cases, in the OR in about 40%, and in the AS in less than 10%. REBOA is considered to provide a bridge to definitive hemorrhage control and patients who have received REBOA in the ER should be quickly transferred to the OR or AS for definitive bleeding control procedures. Furthermore, a hybrid theater can be an ideal environment to address these complex situations with the need for more than one therapeutic modality [38].

Since trauma care is multidisciplinary, the discussion regarding “Who should be performing REBOA?” is not central. REBOA should be a team approach involving all members of the trauma team. This will vary in different settings depending on training, experience, availability of trained staff, proper equipment and availability of blood products, and clotting therapy. Each hospital has a responsibility to define the indications, setting, technique, and process, including “When, Who, Where and How.”

One point we would like to discuss is the mentioned rate of complication [39–41]. With an overall rate of almost 14% and 8.5% major complications, it seems to be quite high. In more than 1/3 of the patients in the registry an introducer of ≥ 10Fr was used and many of the recorded complications were associated with these introducers [42]. Except renal failure, the 1/3 of patients with 10FR introducer or bigger account for as many complications as the 2/3 of patients with an introducer of 9Fr or smaller. A review by Borger van der Burg et al. describes iatrogenic injuries related to REBOA 2.6-5.3% [43]. The main problem regarding REBOA complications in the literature is the fact the many older studies do not mention the rate of complication. In the mate- analysis of Manzano Nunez et al., only one study reported complications [44]. Major complications in our study involve more than just “iatrogenic injuries” and therefore we believe that the rate of complication in our report reflects “real world data” of complications beyond the setting of controlled studies or case series. The mentioned rate of renal failure is in line with a study from the American College of Surgeons Trauma Quality Improvement Program data set (ACS-TOIP) having 10.7% of renal failure [45].

### Limitations

Our study has several limitations that should be addressed. First, our data have been partially retrieved retrospectively and we have a relatively small sample size. All results should therefore be interpreted carefully. Second, this is an international study with limited control over the inclusion and exclusion criteria. This raises the risk of selection bias in the studied population. Third, the registry includes patients in whom REBOA was successfully established and not those in whom REBOA failed. As a major limitation, we are not able to give a number of patients in whom REBOA was attempted but failed. Fourth, the ABOTrauma registry was designed to capture REBOA-specific data and not evaluate the use of the technique in individual cases. Accordingly, indications for and the efficacy of REBOA use are diverse. Fourth, the registry does not capture data regarding the training level of the REBOA performing physician or surgeon. Fifth, there was some missing data, which could have influenced the results; especially the time from admission to REBOA was missing in a relevant number of cases. Finally, the size of the catheters could not be standardized between 22 centers from 13 countries over a 6-year period. There have been fast-changing EVTM techniques with new and smaller catheters in the last few years. The variations in the catheter size may have contributed to different outcomes.

## Conclusion

Using data from the unique ABOTrauma registry, the current study shows that a substantial number of both surgical and non-surgical medical disciplines are successfully performing REBOA to an almost equal extent. Surgical cutdown as an access to the CFA is used less frequently compared with reports in the older literature and puncture of the CFA with the use of external anatomic landmarks and palpation alone is used with a high success rate. With this knowledge, the discussion, “Who should be performing REBOA?” should end. The discussion and future research should instead focus on “Which patients benefit most from REBOA?” and “What level of training is needed to successfully perform REBOA in time-critical situations?” It is the responsibility of each trauma center to define the indications, setting, technique, and process including “When, Who, Where and How” for potential REBOA patients.

## Supplementary Information

**Additional file 1:** **Table S1.** Medical discipline and where REBOA was performed. **Table S2.** Medical discipline and how REBOA was performed.

## Data Availability

The dataset generated and analyzed during the current study is not publicly available due to the ownership of the ABOTrauma Registry but is available from the corresponding author on reasonable request.
