# Supplementary material for: Successfully REBOA performance: does medical specialty matter? International data from the ABOTrauma Registry
Source: World J Emerg Surg. 2020 Nov 23;15:62. doi: 10.1186/s13017-020-00342-z (PMC7685615; doi:10.1186/s13017-020-00342-z)
Supplement: Supplementary file 1 — Additional file 1: Table S1. Medical discipline and where REBOA was performed. Table S2. Medical discipline and how REBOA was performed. [file 13017_2020_342_MOESM1_ESM.docx]

**Table S1:** Medical discipline and where REBOA was performed

| **Medical discipline (N)** | **Age**  **% male** | **ISS** | **ER** | **OR** | **AS** | **Unknown** |
| --- | --- | --- | --- | --- | --- | --- |
| Emergency  Physician (76) | 60 (10-96)  67% | 41  (11-75) | 38  (50%) | 21  (27.5%) | 8  (10.5%) | 9  (12%) |
| Trauma  Surgeon (70) | 38 (10-88)  68.5% | 27  (16-75) | 23  (33%) | 46  (66%) | 1  (1%) | 0 |
| Vascular  Surgeon (54) | 32 (12-85)  85% | 38  (14-75) | 31  (57.5%) | 21  (39%) | 1  (1.75%) | 1  (1.75%) |
| Radiologist  (24) | 51.5 (19-88)  71% | 41  (16-64) | 11  (46%) | 7  (29%) | 5  (21%) | 1  (4%) |
| Anesthetist  (14) | 60 (23-77)  71% | 50  (16-75) | 11  (78.7%) | 1  (7.1%) | 1  (7.1%) | 1  (7.1%) |
| General  Surgeon (9) | 37 (24-90)  89% | 48  (37-57) | 6  (67%) | 2  (22%) | 1  (11%) | 0 |

**Table S2:** Medical discipline and how REBOA was performed

| **Medical discipline** | **N (%)** | **Blind**  **N (%)** | **Ultra sound**  **N (%)** | **Cut down**  **N (%)** | **Fluoro-scopy**  **N (%)** | **Unknown** |
| --- | --- | --- | --- | --- | --- | --- |
| Emergency  Physician | 76  (31%) | 57  (75%) | 12  (15.7%) | 1  (1.3%) | 2  (2.7%) | 4  (5.3%) |
| Trauma  Surgeon | 70  (28%) | 12  (17.2%) | 13  (18.5%) | 31  (44.3%) | 0 | 14  20% |
| Vascular  Surgeon | 54  (22%) | 24  (44.5%) | 7  (13%) | 18  (33.5%) | 2  (4%) | 3  (5%) |
| Radiologist | 24  (9.5%) | 13  (54.2%) | 7  (29.2%) | 0 | 4  (16.6%) | 0 |
| Anesthetist | 14  (5.5%) | 9  (64%) | 5  (36%) | 0 | 0 | 0 |
| General  Surgeon | 9  (4%) | 2  (22.3%) | 1  (11.1%) | 5  (55.5%) | 0 | 1  (11.1%) |
